# Early Predictor Tool of Disease Using Label-Free Liquid Biopsy-Based Platforms for Patient-Centric Healthcare

**DOI:** 10.3390/cancers14030818

**Published:** 2022-02-06

**Authors:** Wei Li, Yunlan Zhou, Yanlin Deng, Bee Luan Khoo

**Affiliations:** 1 Department of Biomedical Engineering, City University of Hong Kong, Hong Kong 999077, China; weili229-c@my.cityu.edu.hk (W.L.); yanlin.deng@my.cityu.edu.hk (Y.D.); 2 Hong Kong Center for Cerebro-Cardiovascular Health Engineering (COCHE), Hong Kong 999077, China; 3 Department of Clinical Laboratory, Xinhua Hospital, Shanghai Jiaotong University School of Medicine, Shanghai 200092, China; 4 Department of Precision Diagnostic and Therapeutic Technology, City University of Hong Kong Shenzhen-Futian Research Institute, Shenzhen 518057, China

**Keywords:** label free, algorithmic analysis, disease prognosis, personalized patient care, patient-derived cell clusters

## Abstract

**Simple Summary:**

We proposed a comprehensive early prediction tool based on liquid biopsy for the label-free phenotypic analysis of cell clusters from clinical samples (*n* = 31). Our custom algorithm analysis, combined with a microfluidic-based tumor model, was designed to assess and stratify cancer patients in a label-free, cost-effective, and user-friendly way. Multiple quantitative phenotypic parameters (cluster size, thickness, roughness, and thickness per area) were derived from the profiling of the patient-derived cell clusters. Our platform could distinguish healthy donors from pretreatment cancer patients with high sensitivity (91.16 ± 1.56%) and specificity (71.01 ± 9.95%). In addition, the ratio of normalized gray value to cluster size (RGVS) parameter was significantly correlated to treatment duration and cancer stage. In conclusion, our patient-centric, early prediction tool will allow the prognosis of patients in a relatively less invasive manner, which can help clinicians identify diseases or indicate the need for new treatment strategies.

**Abstract:**

Cancer cells undergo phenotypic changes or mutations during treatment, making detecting protein-based or gene-based biomarkers challenging. Here, we used algorithmic analysis combined with patient-derived tumor models to derive an early prediction tool using patient-derived cell clusters from liquid biopsy (LIQBP) for cancer prognosis in a label-free manner. The LIQBP platform incorporated a customized microfluidic biochip that mimicked the tumor microenvironment to establish patient clusters, and extracted physical parameters from images of each sample, including size, thickness, roughness, and thickness per area (*n* = 31). Samples from healthy volunteers (*n* = 5) and cancer patients (pretreatment; *n* = 4) could be easily distinguished with high sensitivity (91.16 ± 1.56%) and specificity (71.01 ± 9.95%). Furthermore, we demonstrated that the multiple unique quantitative parameters reflected patient responses. Among these, the ratio of normalized gray value to cluster size (RGVS) was the most significant parameter correlated with cancer stage and treatment duration. Overall, our work presented a novel and less invasive approach for the label-free prediction of disease prognosis to identify patients who require adjustments to their treatment regime. We envisioned that such efforts would promote the management of personalized patient care conveniently and cost effectively.

## 1. Introduction

Cancer is one of the leading causes of mortality globally [1,2]. The conventional diagnostic method for cancer is solid tumor biopsy, which is invasive and can cause discomfort. Besides, the procedure is very cumbersome and time consuming. Liquid biopsy provides a relatively less invasive method for detecting disease-related biomarkers, leading to new technologies [3,4]. The advantages of liquid biopsies, such as ease of sample collection and minimal invasiveness, make it an ideal method for routine evaluation. Common biomarkers in liquid biopsy can be protein, gene, or cell based. Detecting proteins or genes involves targeted probe labeling, which requires a priori knowledge of a comprehensive biomarker profile. However, due to the heterogeneity of tumors, common protein and gene cancer-associated biomarkers cannot fully recapitulate the characteristics of tumors [5].

Conventional cancer-related research usually utilizes commercially available cancer cell lines; however, these are not clinically relevant, and are limited for applications such as anti-cancer drug screening, preclinical testing, and biomarkers discovery [6]. On the other hand, patient-derived tumor models can effectively promote translational efforts. Patient-derived tumor models can be classified into five subtypes, i.e., 3D culture systems, conditionally reprogrammed cell cultures, organotypic tissue slices, patient-derived xenograft models, and microchamber cultures [7]. Three-dimensional cultures are generally preferred, as they can better recapitulate the in vivo environment, and hence demonstrate higher sensitivity to drug treatment, as well as reflect biomarker profiles more similar to in vivo environments than 2D cultures [8].

Effective early prediction tools for personalized medicine should consider the following factors: (i) strong correlation with the disease, (ii) timely readouts, and (iii) ease of use. These factors are critical for clinicians to understand the patient’s condition and design appropriate treatment measures. Current cancer-associated algorithms focus on analyzing non-clinical spheroid characterization [9,10]. Here, we developed a novel, label-free analysis tool based on patient-derived tumor models from liquid biopsy (LIQBP) for the early prediction of disease prognosis. The tumor models comprised heterogeneous clusters containing circulating tumor cells and immune cells unique to each patient, and could be established within the duration of approximately one treatment cycle (i.e., 14 days), allowing rapid intervention by clinicians based on LIQBP readouts. Our LIQBP platform analysis parameters were based on four core aspects: size, thickness, roughness, and thickness per area (TA). We demonstrated that the cluster phenotypes from pretreatment patient samples (*n* = 4) were distinct from those established with healthy controls (*n* = 5). The LIQBP could also distinguish response subtypes (e.g., treatment time point, tumor type). Compared with other parameters, cluster TA was the most stable phenotypic parameter. It was strongly correlated to the cancer stage and treatment duration of patients with gastric cancer (*n* = 12) and breast cancer patients (*n* = 10).

Due to its relatively lower invasiveness and cost, liquid biopsy with the LIQBP platform has multiple advantages over conventional patient response assessments and facilitates routine screening. We have also built a fully automated interface for user-friendly and robust operations in clinics. We envisioned that the LIQBP platform could become a powerful predictor tool for patients, enabling routine monitoring and developing therapeutic guidelines.

## 2. Results

### 2.1. Establishment of a Patient-Derived Tumor Model from Liquid Biopsy (LIQBP) to Evaluate Clinical Prognosis

**Figure 1:**
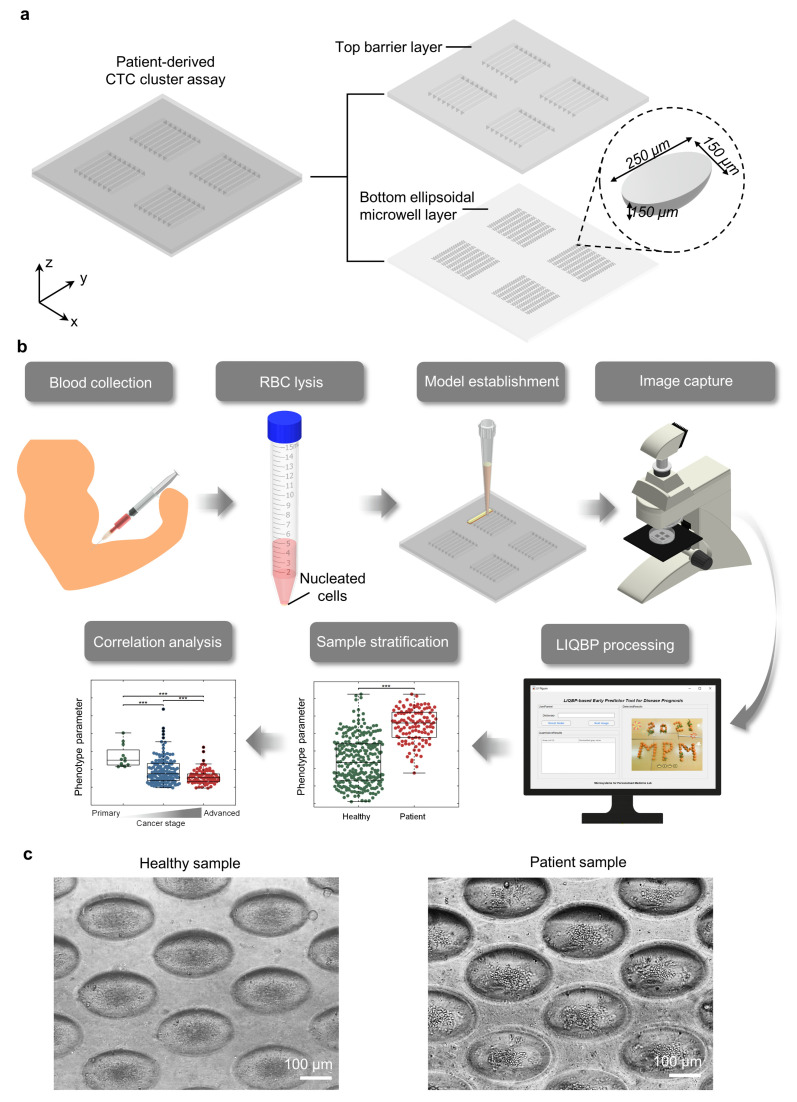
Using a patient-derived tumour model from a liquid biopsy (LIQBP) with multiple phenotyping for label-free prediction of disease prognosis. (**a**) Schematic illustration of the multiplexed tumor model with four units. The model was established within a microfluidic device with a top barrier layer and a bottom ellipsoidal microwell layer. Each array had eight channels comprising 300 microwells each. (**b**) Workflow for the establishment of tumor models. Peripheral blood was collected from the vein of the patient. The blood sample was lysed to remove red blood cells, and the remaining nucleated cell fraction was seeded for culturing over 14 days. Images of cultured cells were captured by a phase-contrast microscope and analyzed via LIQBP for sample stratification, with a clear distinction between patient and healthy samples. The clusters could be classified as four core phenotypes, covering the parameters of cluster size, thickness, roughness, and TA. *** Represents *p* ≤ 0.001. (**c**) Representative grayscale images to demonstrate the distinct morphological differences between clusters established from healthy donors (**left**) and cancer patients (**right**). Scale bar, 100 μm.

To develop a clinically relevant point-of-care system for the routine evaluation of patient prognosis, we established a patient-derived tumor model from liquid biopsy using a microfluidic-based biochip. The tumor models could be derived from liquid (blood) biopsy within 14 days and consisted of two parts: (i) a bottom, ellipsoidal-shaped, tapered microwell layer, allowing the different components of the co-cultures to interact with one another for cell cluster establishment, and (ii) a top barrier layer to retain fluids and to avoid mixing between channels (Figure 1a). The length, width, and depth of each microwell were 250 μm, 150 μm, and 150 μm, respectively.

Peripheral circulating blood was collected and lysed to remove red blood cells (Figure 1b). The remaining nucleated cells were suspended and seeded into the chip (see Materials and Methods). The cultures established from healthy people and patients were distinctly different in morphology (Figure 1c) and were used in our analysis for automated classification and prediction. The cultures of healthy samples would generate irregular and loose monolayers of cellular debris or residual blood cells [11]. In contrast, clusters formed with patient samples were rough, comprising a heterogeneous mixture of blood cells and cancer cells.

### 2.2. An Automated Bioinformatic Analysis Tool to Achieve Label-Free Prediction of Disease Prognosis with Cluster-Based Clinical Phenotyping

Current cancer-related algorithms focus on characterizing tumor models established from cell lines. We developed a novel, label-free analysis tool that can perform an extensive analysis of patient subtypes and facilitate routine screening based on tumor models derived from the liquid biopsy of patients to predict early-stage disease and to reflect disease prognosis.

**Figure 2:**
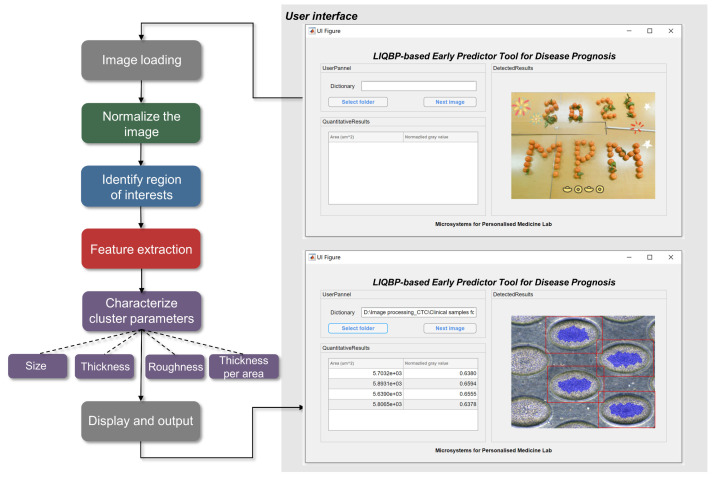
Procedures of LIQBP with multiple phenotypes analysis. User interfaces and the workflow of LIQBP with label-free phenotyping analysis: the image was normalized by flat-field correction; regions of interest (ROI) were identified sequentially; cluster size and thickness were displayed and saved for further analysis.

The integrated LIQBP platform included a user interface and a user-friendly program based on flat-field correction, auto ellipse detection, edge detection, and a morphology algorithm. The algorithm consisted of four core steps (Figure 2): (1) Normalizing images through flat-field background correction to achieve a uniformly illuminated image; (2) Using the auto-ellipse detection algorithm to identify the regions of interest (ROIs; microwells). Images were cropped to the tangent rectangles of the resultant ellipses that corresponded to the microwell locations; (3) Feature extraction to identify the cropped regions of interest (cROIs; clusters) within the microwells, using an edge detection-based algorithm for locating, dilating, eroding, and binarizing the cluster region; (4) Parameter characterization, including cluster size, thickness, roughness, and TA.

The cluster thickness was determined according to the normalized gray value (nGV), representing the amount of light transmitted through the cell cluster. To eliminate fluctuations in the maxima and minima of gray values due to the imaging technique or microscope variation, the nGV was obtained relative to the maxima obtained from each microwell. An nGV approaching 0 would reflect the presence of clusters with thicker cell layers.

The cluster roughness was defined by the standard deviation of the gray value (SD^GV^). If the SD^GV^ was large, the surface of the cluster was rough, reflecting the presence of cell clusters formed from patient samples. A higher normalized SD^GV^ (nSD^GV^) would reflect increased heterogeneity within the clusters due to the presence of tumor-associated immune cells. The cluster TA was determined by the ratio of the nGV to the cluster size (RGVS). A lower RGVS correlated with the presence of thicker clusters within the area.

To utilize LIQBP and obtain the quantitative attributes of the clusters, the user would first select the file dictionary of the test image. The parameter outputs would be automatically obtained. Specifically, we first performed flat-field background correction on the test image(s) (Figure S1a). The Gaussian filter was used to extract the background signal, and the background signal was then subtracted to normalize the test image. Then, an auto ellipse detection algorithm was executed to locate the regions of interest (ROI; e.g., microwell) for cropping and subsequent processing.

To identify cell clusters in the ROI, the LIQBP involved the automatic cropping of images to display individual microwells (Figure S1b). Since cell clusters were a heterogeneous mixture of tumor-associated immune cells, the white pixels (space within the clusters) in the binary image were filled to generate closed polygons. The connected objects on the image boundary were removed to remove noise pixels, and the image was eroded multiple times using linear structure elements. The white pixels in the binary image were identified as the region of interest (ROI; clusters).

The number of white pixels *N_w_* and the total number of pixels *N_t_* were determined using the processed binary images (Figure S1c). Following which, as the length and width of each microwell were fixed, the size of the cell cluster could be obtained, as stated in Equation (1):

(1) $$S=\frac{Nw}{Nt}\times Lm\times Wm$$

where *S* represented the cluster size, *L_m_* and *W_m_* were constants (250 μm and 150 μm) and represented the length and width of the microwells, respectively.

The final processed binary images were converted to grayscale, and the gray values were obtained to extract the cell cluster area (Figure S1d). Regions with higher gray values indicated the presence of more transmitted light (less or no cells).

### 2.3. Clinical Validation with LIQBP

**Figure 3:**
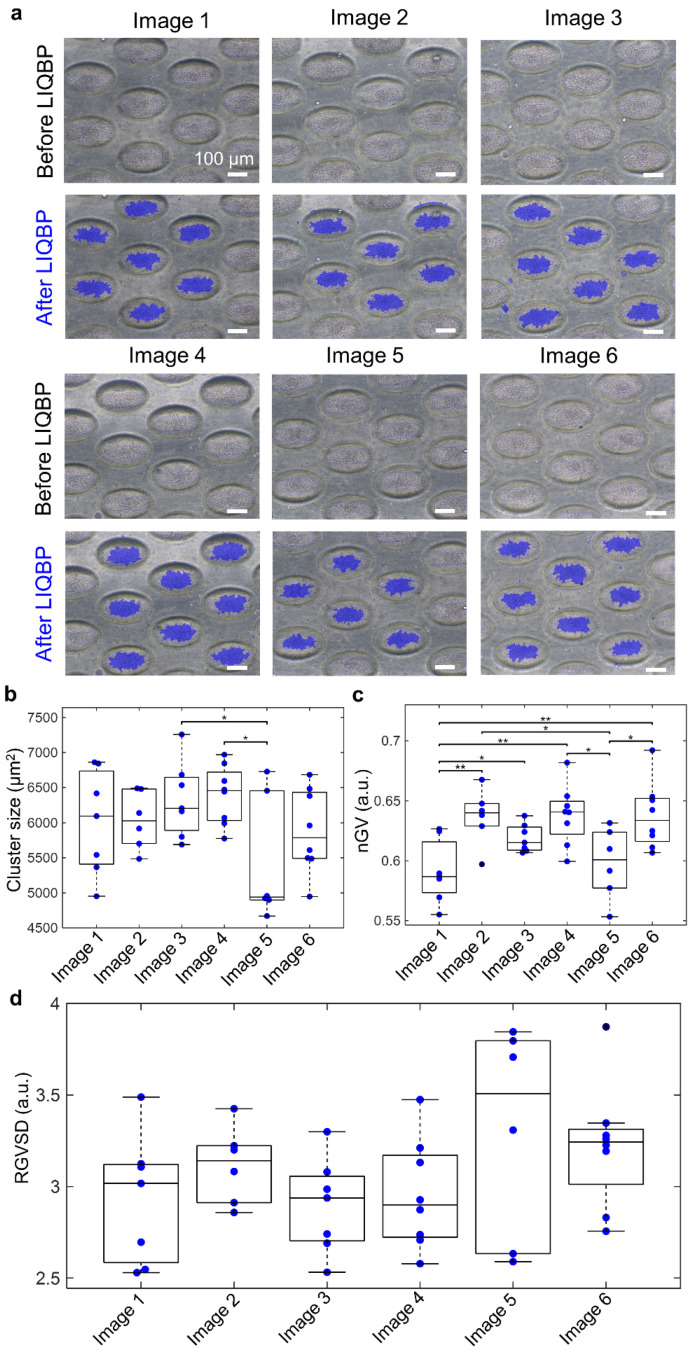
Robustness validation of LIQBP. (**a**) Representative images before the LIQBP and their corresponding detected images after the LIQBP. These images are cell clusters cultured from one patient sample. Blue shades in the detected images represented the detected patient-derived cell cluster. Scale bar, 100 μm. (**b**) Boxplot of the size of patient-derived cell clusters. (**c**) Boxplot of nGV of patient-derived cell clusters. ** Represents *p* ≤ 0.01 and * represents *p* ≤ 0.05. (**d**) Boxplot of RGVS of patient-derived cell clusters. RGVS values across clusters of the same patient remained relatively constant.

To validate the clinical utility of the LIQBP, we obtained images of clusters derived from clinical samples. The expression level of cancer-associated biomarkers was heterogeneous across the samples, as previously reported [11]. The patient-derived cultures comprised multilayered cancer cells and tumor-associated immune cell clusters [11]. We first confirmed the robustness of the LIQBP by processing the images of clusters derived from the same patient sample and determined the cluster size, nGV, and RGVS (Figure 3a). Patient samples were of a heterogeneous composition, as reflected by their slight variation in cluster size and nGV (*p* > 0.01) (Figure 3b,c). However, the parameter RGVS was relatively constant across clusters of the same sample (Figure 3d), validating the robustness of the LIQBP for the evaluation of clinical samples.

### 2.4. Distinct Stratification of Healthy and Patient Phenotypes with LIQBP

To evaluate the performance of our proposed method when classifying healthy donors and cancer patients, we first collected peripheral blood samples (*n* = 9) from healthy people (*n* = 5) (Table 1; no. 1–5) and cancer patients (pretreatment; *n* = 4) (Table 1; no. 10, 15, 16, and 27) for culturing and to perform subsequent phenotypic analysis.

**Figure 4:**
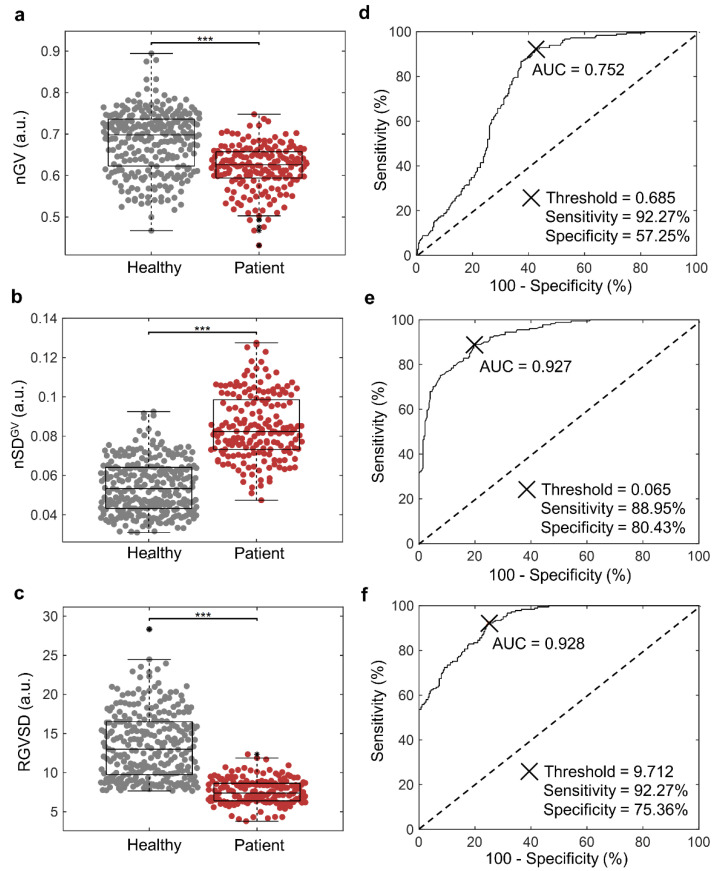
Patient stratification of healthy and cancer patients with LIQBP. (**a**–**c**): Boxplots illustrating correlation analysis of nGV (0.68 ± 0.07 and 0.62 ± 0.05), nSDGV (0.054 ± 0.013 and 0.085 ± 0.017) and RGVSD (13.50 ± 4.22 and 7.60 ± 1.57) for healthy and patient cohorts, respectively. *** Represents *p* ≤ 0.001. (**d**–**f**): Corresponding ROC analysis for (**a**–**c**), respectively. The resultant AUC, threshold, sensitivity, and specificity analyses are as shown in the plots.

Based on images obtained from healthy and patient sample cohorts, we determined nGV, nSD^GV^, and the ratio of the normalized gray value to the normalized standard deviation of clusters (RGVSD) (Figure 4a–c, respectively). High nGVs (>0.685) reflected the presence of thin clusters. We demonstrated that, compared with the healthy cohort, the clusters from the patient cohort have significantly lower nGVs, higher nSD^GV^ values, and lower RGVSD values (nGVs^−^/nSD^GV+^/RGVSD^−^), reflecting the presence of thick and rough clusters. The RGVSD parameter was the most successful at stratifying between patient and healthy cohorts.

Next, we performed receiver operating characteristic (ROC) analysis by obtaining the area under the curve (AUC) (Figure 4d–f). We determined the AUCs of nGV, nSD^GV^, and RGVSD to be 0.752, 0.927, and 0.928, respectively. The RGVSD yielded the highest AUC, demonstrating that the RGVSD had the best performance for distinguishing between healthy donors and cancer patient cohorts. The thresholds were determined by Youden’s index, which maximizes the sum of sensitivity and specificity. We demonstrated that the LIQBP has a sensitivity and specificity of 92.27% and 57.25% to distinguish healthy and patient samples based on cluster thickness at the threshold setting of 0.685. The sensitivity and specificity of LIQBP based on cluster roughness were significantly improved to 88.95% and 80.43%, respectively, at the threshold of 0.065. The sensitivity and specificity of LIQBP based on RGVSD were 92.27% and 75.36%, respectively, at the threshold of 9.712.

Overall, the high AUC (0.869 ± 0.083), sensitivity (91.16 ± 1.56%), and specificity (71.01 ± 9.95%) of these parameters validated the use of thickness, roughness, and RGVSD as efficient indexes for distinguishing between the healthy donor and cancer patient cohorts. Based on the thresholds (nGV: <0.685, nSD^GV^: >0.065, and RGVSD: <9.712), the patient cohort was labeled and further analyzed with another three parameters (size, nGV, and RGVS) to reflect cluster size, thickness, and TA for correlation with clinical parameters (treatment cycles and cancer staging).

### 2.5. Correlation of RGVS Parameter with Treatment Cycles

To study the correlation between cluster parameters and patient prognosis, we analyzed clinical samples of liquid (blood) biopsy from cancer patients throughout their treatment (*n* = 22) (Table 1; no. 6–22, 27–31). Four samples with non-optimal optical images were not included in the analysis. Patients underwent four treatment cycles, namely, pretreatment (*n* = 4), treatment cycle 1 (*n* = 9), treatment cycle 3 (*n* = 3), treatment cycle 4 (*n* = 2), treatment cycle 5 (*n* = 2), and treatment cycle 6 (*n* = 2). Parameters, including the cluster size, nGV, and RGVS, were determined.

**Figure 5:**
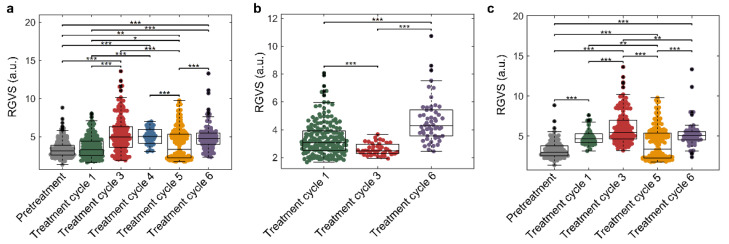
Clinical correlation between patient-derived cell cluster parameters and treatment cycle number. (**a**) Boxplot of RGVS against treatment cycle number, based on samples (Table 1; no. 6–22, 27–31; *n* = 22) obtained at pretreatment (3.45 ± 1.15), treatment cycle 1 (3.63 ± 1.31), treatment cycle 3 (5.29 ± 2.27), treatment cycle 4 (5.08 ± 1.02), treatment cycle 5 (4.02 ± 2.02), and treatment cycle 6 (4.97 ± 1.66). (**b**) Boxplots of RGVS against treatment cycle number, based on gastric cancer samples (*n* = 8) obtained at pretreatment (3.35 ± 1.21), treatment cycle 1 (2.61 ± 0.42), and treatment cycle 3 (4.67 ± 1.60). (**c**) Boxplots of RGVS against treatment cycle number, based on breast cancer samples (*n* = 10) obtained at pretreatment (3.26 ± 1.08), treatment cycle 1 (4.87 ± 1.01), treatment cycle 3 (6.04 ± 2.00), treatment cycle 5 (4.02 ± 2.02), and treatment cycle 6 (5.23 ± 1.68). *** Represents *p* ≤ 0.001, ** represents *p* ≤ 0.01, and * represents *p* ≤ 0.05.

We demonstrated that for samples in the advanced stages of treatment, the cluster size was progressively and significantly smaller (*p* < 0.001) than the samples derived before treatment, with significantly smaller clusters obtained from samples after prolonged treatment (after three cycles of treatment; Figure S2a). The nGV fluctuated as the treatment cycles increased (*p* < 0.001), reflecting the heterogeneity of cluster thickness on patient treatment responses (Figure S2b). Since cancer is a dynamic and heterogeneous disease, tumor and tumor-associated cells exhibit distinct molecular signatures, leading to varied responses under treatment [12,13,14], which were reflected in the morphological differences observed among the clusters. On the other hand, RGVS was positively correlated with the number of treatment cycles (Figure 5a). There was no significant difference for the RGVS of clusters from samples of pretreatment and treatment cycle 1. However, as the treatment time increased, the RGVS of the samples from prolonged treatment increased significantly (after three cycles of treatment; *p* < 0.001), reflecting the decrease in cluster TA.

To further analyze the correlation between patient-derived cell clusters and treatment cycles with a specific cancer type, we obtained cluster size, nGV, and RGVS from the gastric (*n* = 8) (Table 1; no. 6–9 and 19–22) and breast cancer cohorts (*n* = 10) (Table 1; no. 10–14 and 27–31). The cluster size of the samples from treatment cycle 6 was significantly smaller (*p* < 0.001) than the primary treatment cycles (Figure S3). The RGVS in treatment cycle 6 was significantly higher (*p* < 0.001) than the values obtained in cycles 1 and 3, which verified the significant positive correlation of RGVS with treatment cycles (Figure 5b). Similarly, for the breast cancer cohorts, the cluster size of samples from patients during all the treatment cycles was significantly smaller (*p* < 0.001) than those obtained before treatment, and the nGV values of samples from treatment cycles 3 and 5 were significantly higher (*p* < 0.001) than those of the samples from cycle 1 (Figure S4).

However, the correlation of RGVS with the treatment cycle number was highly significant (*p* < 0.001) for the breast cancer cohorts (Figure 5c). Specifically, the RGVS of clusters increased in all the treatment cycles for treated samples compared to pretreatment samples, indicating looser clusters due to treatment.

### 2.6. Correlation of RGVS Parameter with Cancer Staging (TNM Staging)

To analyze the correlation of patient-derived cell clusters with TNM staging, we obtained the three parameters, i.e., cluster size, nGV, and RGVS, from the clinical samples (Table 1; no. 6–22 and 28–31), and analyzed their correlation with T staging, N staging, and cancer staging. A breast cancer sample with unknown TNM and cancer staging was not included in the analysis (Table 1; no. 27).

**Figure 6:**
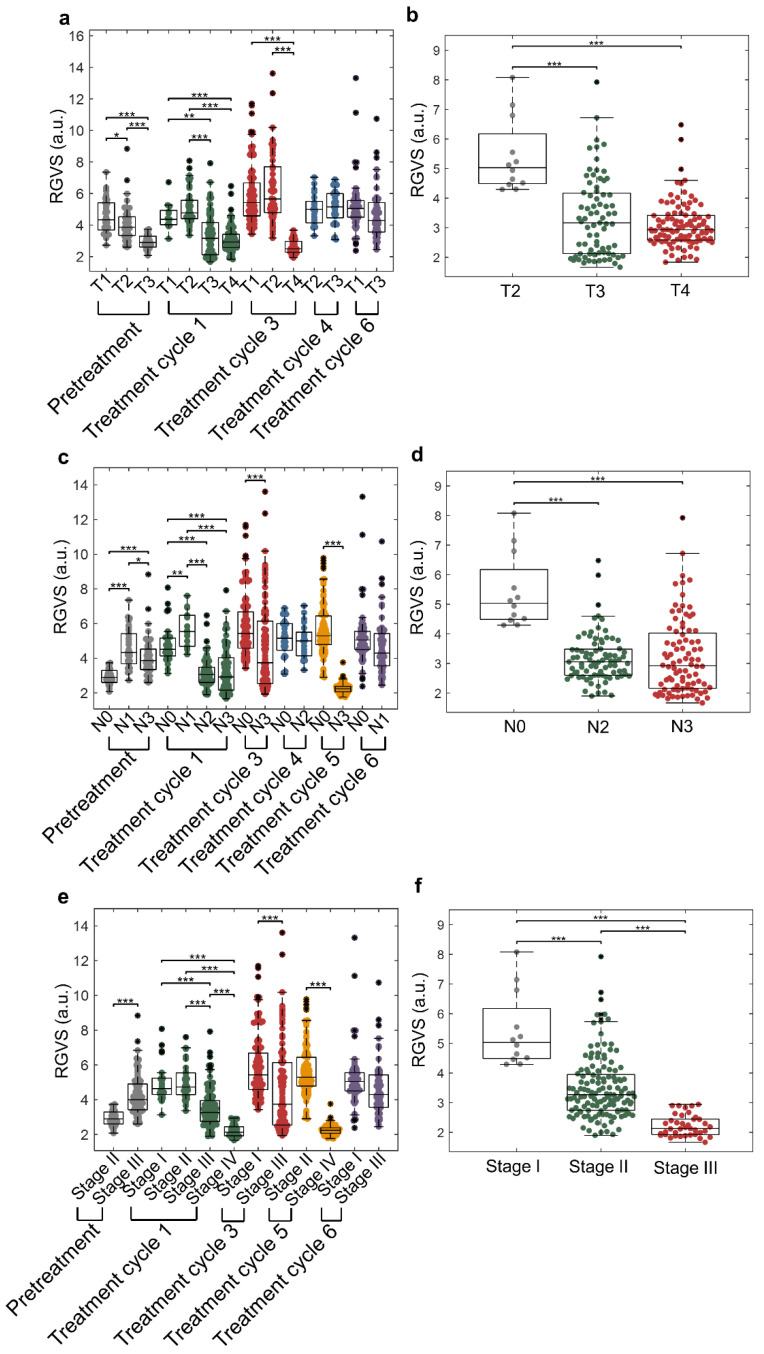
Clinical correlation analysis of patient-derived cell clusters with cancer staging. (**a**,**b**) Boxplot of the RGVS of patient clusters versus T staging from 19 clinical samples (pretreatment (*n* = 3), treatment cycle 1 (*n* = 9), treatment cycle 3 (*n* = 3), treatment cycle 4 (*n* = 2), and treatment cycle 6 (*n* = 2)) and gastric cancer samples in treatment cycle 1 (Table 1; no. 6–9, 21–22; *n* = 6). (**c**,**d**) Boxplot analysis of patient clusters versus N staging from 21 clincal samples (pretreatment (*n* = 3), treatment cycle 1 (*n* = 9), treatment cycle 3 (*n* = 3), treatment cycle 4 (*n* = 2), treatment cycle 5 (*n* = 2), and treatment cycle 6 (*n* = 2)) and gastric cancer samples in treatment cycle 1 (*n* = 6). (**e**) Boxplots of the RGVS of patient clusters versus cancer stages (stage I to stage IV) from 19 clincial samples (pretreatment (*n* = 3), treatment cycle 1 (*n* = 9), treatment cycle 3 (*n* = 3), treatment cycle 5 (*n* = 2), and treatment cycle 6 (*n* = 2)). (**f**) Boxplots of the RGVS of patient clusters versus cancer stages (stage I and stage III) from gastric cancer patients (*n* = 6) in treatment cycle 1, respectively. *** Represents *p* ≤ 0.001, ** represents *p* ≤ 0.01, and * represents *p* ≤ 0.05.

T staging describes the size of the primary tumor. We demonstrated that the RGVS decreased significantly for the T1 to T3 samples obtained from patients before treatment (*p* < 0.05), and a similar trend could be observed from T2 to T4 for the patient cohort under treatment cycle one and treatment cycle 3 (*p* < 0.001), which indicated that RGVS was negatively correlated with T staging. The clusters were thicker per area for patients with more advanced T staging than those under preliminary T staging (Figure 6a). We further analyzed the correlation of cluster parameters against T staging with specific patient cohorts (gastric cancer). For patients with gastric cancer under T2 to T4 under all treatment time points (Table 1; no. 6–9, 19–22), the resultant RGVS significantly decreased (*p* < 0.001) (Figure S5). Similar decreasing trends (*p* < 0.001) were observed with clusters derived from gastric cancer samples under treatment cycle I (Table 1; no. 6–9, 21–22; *n* = 6) (Figure 6b).

N staging described the degree of regional lymph nodes metastasis. We demonstrated that the RGVS correlated significantly (*p* < 0.005) with specific N staging (e.g., from N0 to N3 in treatment cycles 1, 3, and 5) (Figure 6c). Interestingly, for gastric cancer samples under all treatment cycles, RGVS was maintained at high values at preliminary N staging (N0 and N1) and decreased significantly to a low level in advanced N staging (N2 and N3) (*p* < 0.001), which verified an inverse correlation of RGVS to N staging, and clusters with advanced N staging tended to be thicker within each cluster area (Figure S6). However, for gastric cancer samples obtained from patients under treatment cycle 1, RGVS only correlated significantly with specific N staging (*p* < 0.001) (Figure 6d).

We also demonstrated that the RGVS of clusters was decreased significantly at more advanced overall cancer staging, specifically from stage III to IV in treatment cycle 1, stage I to III in treatment cycle 3, and stage II to IV in treatment cycle 5 (*p* < 0.001) (Figure 6e). A similar significant decreasing trend for RGVS was observed in all the gastric cancer samples (*p* < 0.001) (Figure S7) and gastric samples under treatment cycle I (*p* < 0.001) (Figure 6f). In short, the results verified that RGVS correlated inversely with cancer staging, including T staging, N staging, and overall cancer staging, and the capability of RGVS to distinguish cancer patients in different cancer staging significantly.

## 3. Discussion

In recent years, microfluidic technology has been widely used in the field of particle detection [15] and biomedicine, such as with point of care testing [16,17,18,19], organs-on-a-chip [20,21], drug discovery [22], and liquid biopsy [23,24,25,26]. Cell sorting techniques can be divided into label-based and label-free technology [27,28]. Label-based methods generally rely on affinity binding technology or the use of different optical, acoustic, electrical, or magneto-caloric properties between cancer cells and blood cells to identify biomarkers. Label-free methods mainly capitalize on the unique physical properties of cancer cells, such as size, density, stiffness, viscosity, and deformability. Although label-free methods tend to achieve high-throughput separation and detection, drawbacks, such as biofouling, low recovery rates, and loss of cell viability, are still prevalent.

Compared with other tumor models (Table S1), our platform demonstrated three key advantages as a unique screening tool: (i) The analysis was based on the establishment of patient-derived clusters from liquid (blood) biopsy. The samples were highly heterogeneous because they were a mixture of circulating tumor cells, white blood cells, and platelets. Tumor models based on cancer cell lines cannot fully recapitulate the conditions in vivo. Their homogeneity renders it much easier to interpret with standard algorithms; (ii) It is a method of utilizing phase-contrast microscopy to obtain morphological features, promoting ease of capture and low cost for routine screening. Quantitative readouts could also be obtained; (iii) Cell-based algorithms were usually classified as physics- and data-driven-based analyses. Physics-driven algorithms focus on specific phenotypes or features of cell cultures. However, we uniquely derived multiple parameters (e.g., cluster size, nGV, and RGVS) for a more comprehensive study to derive the correlation between cluster phenotypes and clinical parameters, including treatment cycles and cancer staging.

On the other hand, data-driven analyses are mainly based on machine learning and deep learning techniques. They usually need many data sets and prolonged periods of training. In contrast, our LIQBP could provide a multiparameter analysis, including morphological features, such as cluster edge detection, making it robust and compatible for use with a range of microscopy magnification. Our customized user interface also allowed the real-time display of processed images, simplifying the process of analysis and interpretation.

Clinical samples were cultured with high viability (92.57 ± 8.85%) in each microwell (Figure S8), and cohorts could be distinctly stratified with high sensitivity and specificity. We demonstrated that the cluster size correlated significantly with the number of treatment cycles and cancer staging, while nGV correlated with treatment durations and cancer staging. However, compared with cluster size or nGV alone, RGVS was validated as a robust and comprehensive parameter to stratify between healthy and patient cohorts or reflect outcomes at a particular treatment stage and for TNM staging.

Due to the multivariate factors affecting tumor progression, it has been challenging to establish a clinically relevant cancer model in vitro. Multivariate factors include tumor growth, proliferation, migration, invasion, matrix remodeling, dormancy, infiltration, extravasation, angiogenesis, and drug delivery [29]. In addition, tumors are highly heterogeneous structures, including cancer and non-cancerous cells, which is rarely reflected in vitro models [29]. Here, we described a label-free predictive tool for disease prognosis using patient-derived tumor models from the liquid biopsy. The LIQBP integrated an interface and the label-free image analysis program, which could be customized to add or remove functions, providing ease of operation and flexibility in applications. Test images could be analyzed in batches within a minute, significantly reducing manpower requirements and the speed at which treatment intervention could be realized. The LIQBP could provide readouts in a label-free and quantitative manner without the need for visualization. As such, the low cost, minimal training, and no associated toxicity of dyes render LIQBP a highly beneficial prediction tool for use even in regions with limited resources.

The LIQBP was able to significantly and robustly reflect disease heterogeneity among cancer types. For example, the fluctuations of nGV were more distinct in breast cancer cohorts than the gastric cancer cohorts, which could be due to the highly heterogeneous nature of breast cancer. Briefly, breast cancer patients can be classified as multiple molecular subtypes (e.g., luminal A (ER+/PR+ and Ki67-low), luminal B (ER+/PR+ and HER2+ or HER2–, and Ki67-high), HER2-enriched (HER2+), normal breast-like, basal-like, and claudin-low) [30]. The differences between patients lead to intertumoral heterogeneity, which would affect patient diagnosis, treatment, and prognosis. Intratumoral heterogeneity on tumor cell subpopulations within the breast primary tumor and metastases could also trigger diversity in response [31].

With the development of smartphone-based biosensors, optical systems for visualization could also be further minimized to achieve portable on-site detection [32,33]. The tumor model has been previously demonstrated to allow the discovery and validation of new combinatorial drug strategies [34], and efforts are being made to demonstrate the utility of this technique for drug discovery. Furthermore, we could also integrate convolutional neural networks to realize the transition from physical-driven analysis to data-driven analysis and realize high-throughput screening [35,36,37,38]. In addition, we are currently expanding patient cohort size, along with serial sampling to validate the LIQBP platform for clinical utility. The assay was optimized to reflect patient prognosis based on the frequency of cluster formation [11]. Specifically, cluster formation was seen progressively less frequently in blood samples from patients who had undergone longer durations of systemic therapy by at least two fold, as previously reported. The presence of clusters reflected the presence of viable cancer cells, which could be of interest, as they could be correlated to the presence of cells with metastatic potential, which provided this assay with an advantage over genetic analysis.

Overall, the LIQBP platform provided a user-friendly method that was simple and easy to operate, facilitating clinical use for routine screening and rapid intervention. We envision that the LIQBP could have vast applications to decentralize healthcare, improve cancer diagnosis, and promote point-of-care, in-house prognostic support.

## 4. Materials and Methods

### 4.1. Fabrication of the Microfluidic-Based Tumor Model

The integrated microfluidic-based tumor model comprises two polydimethylsiloxane (PDMS) layers assembled via plasma treatment. The master mold with ellipsoidal microwells was fabricated according to the diffuser back-side lithography procedure [39]. The mold contains eight arrays, and each array contains 1000 ellipsoidal microwells. The length, width, and depth of each ellipsoidal microwell are 250 μm, 150 μm, and 150 μm, respectively. The dimensions of the microwells were optimized to generate cell clusters under minimal exposure to shear flow under fluid exchange, as previously reported [11,40].

PDMS (Sylgard 184 Silicone Elastomer Kit, Dow Corning, Midlan, MI, USA) was prepared with the ratio of 10:1 (elastomer versus curing agent). The PDMS was poured for casting patterns from the mold and then put into an oven for baking for 2.5 h at 70 °C. After that, the PDMS with the ellipsoidal microwells pattern was peeled off. The master mold of the barrier layer was fabricated using 3D printing. The PDMS was poured into the PLA mold and baked for 2.5 h at 70 °C. Then, the PDMS was peeled off. The microwell and barrier layers were assembled with plasma treatment for 5 min with 700 mmtor. Finally, the assembled microfluidic chip was put into an oven to bake for 2 h at 70 ℃.

### 4.2. Clinical Samples Preparation

Blood samples were collected from 31 patients (Table 1). The institutional review board approved this study with ethical approval (certificate no. XHEC-NSFC-2020-078). All patients consented to be included in the study. Blood samples were collected at different treatment timing points from each patient. They were collected in EDTA-coated vacutainer tubes (Becton Dickinson) and mixed with red blood cell lysis buffer (Life Technologies) for three to five min at room temperature and then centrifuged at 1000× *g* for five min to remove the supernatant. The lysis reaction was washed with sterile phosphate-buffered saline (PBS) three times.

### 4.3. Cell Seeding

Cell suspension from each tested clinical sample was distributed evenly into the microchannel. The samples were suspended with Dulbecco’s Modified Eagle Medium (DMEM) (10% Fetal Bovine Serum (FBS), 1% penicillin-streptomycin) at 1.6 mL and mixed gently [40]. The cell viability of seeding cells was ~100%, and cell morphology and size were not altered after lysing [41]. Samples equivalent to 200 µL whole blood samples were seeded into the microchannels and kept at 37 °C in 5% CO_2_ under humidified conditions. The concentration was adjusted if the nucleated blood cell count went significantly below average (<1 × 10^5^/mL).

### 4.4. Maintenance of Cell Culture

After cell seeding, the integrated chip was placed in a 150 mm dish and incubated under humidified conditions with 5% CO_2_ and 1% O_2_ at 37 ℃ for 14 days [40]. The media were refreshed every three days, i.e., removing 0.2 mL of old culture medium with a hand pipette and replacing it with 0.2 mL of fresh medium. A syringe pump could be used to remove and inject media at 200 uL/min to maintain the consistency of the cell cultures.

### 4.5. Immunostaining

A cocktail containing Calcein-AM (Invitrogen, #C3100MP, Carlsbad, CA, USA) and SYTOX Red (Invitrogen, # S34859, Carlsbad, CA, USA) was incubated for 30 min at 37 °C to evaluate the viability of cells in the microchannel. The assay was washed gently by PBS and imaged by a confocal laser scanning microscope (Leica TCS SP8 MP, Wetzlar, Germany).

### 4.6. Label-Free Monitoring of Tumor Models

A phase-contrast microscope (Nikon, Eclipse Ci-L, Tokyo, Japan) was used to monitor the cultured results in the integrated chip on the 1st, 3rd, 7th, and 14th days of culture. The exposure time, ISO sensitivity, and white balance of the CCD camera on the microscope were fixed to ensure the same illumination conditions in each experiment.

### 4.7. Image Processing

The customized LIQBP software contained an interface and a label-free image algorithm designed with the MATLAB App Designer. The detected cluster and the quantitative parameters of the clusters’ phenotypes would display automatically on the software. During image processing, background correction was performed to pre-process the tested image. After that, the microwell region was detected, cropped, and saved automatically for further cluster identification.

The original image was converted to grayscale for image analysis for cluster recognition. The Sobel operator detected the edges in the image and converted them into a binary format based on the threshold. Next, the binary edge image was expanded by linear structural elements to enhance the features in the binary image. White pixels within the middle of the binary image indicated the ROIs.

### 4.8. Statistical Analysis

Student’s t-tests were used to evaluate the associations between each independent variable. P values among each group were calculated. The ROC curve was constructed using the nGV, nSD^nGV^, and RGVSD as predictors for distinguishing healthy and patient samples. The cutoff value was obtained using Youden’s index, which maximized the sensitivity and specificity. The sensitivity was determined as the ratio of true positives and the number of true positives plus false negatives. The specificity was determined as the ratio of true negatives and the number of true negatives plus false positives. Triplicates were carried out for all experiments.

## 5. Conclusions

In conclusion, we described a predictive tool that integrated patient-derived tumor models from liquid biopsy and label-free quantitative analysis for personalized cancer diagnosis, rapid screening, and predictive treatment. The predictive tool could evaluate multivariate factors, including cluster size, thickness, roughness, and TA. The cluster TA was strongly correlated to cancer staging and treatment duration among these parameters. LIQBP was validated with gastric and breast cancer clinical samples and can be used in many cancer types due to its label-free requirements. Specific thresholds could be used to establish patient stratification. Overall, our LIQBP tumor model system has the vast potential to develop a wide range of clinical applications and promote decentralized healthcare.

## Figures and Tables

**Table 1:** Clinical demographics of cancer patients. p = pathological, c = clinical, T = size or direct extent of the primary tumor, N = degree of spread to regional lymph nodes, M = presence of distant metastasis.

Sample No.	Cancer Type	TNM Stage	Cancer Stage (0 to IV)	Treatment Cycle	Age	Gender
1	Healthy	-	-	-	48	Male
2	Healthy	-	-	-	65	Female
3	Healthy	-	-	-	62	Male
4	Healthy	-	-	-	36	Female
5	Healthy	-	-	-	39	Male
6	Gastric	pT4N2M0	IIIA	1	69	Female
7	Gastric	pT4N2M0	IIIB	1	69	Male
8	Gastric	pT2N0M0	IB	1	53	Female
9	Gastric	pT3N3M0	IIIB	1	63	Male
10	Breast	pT2N3M0	IIIC	Pretreatment	42	Female
11	Breast	pT2N0M0	IIA	1	64	Female
12	Breast	pT2N1M0	IIB	1	57	Female
13	Breast	pT1N0M0	IA	1	50	Female
14	Breast	cT2N3M0	IIIC	3	49	Female
15	Colon	pT3N0M0	IIA	Pretreatment	67	Female
16	Colon	pT1N1bM0	IIIA	Pretreatment	71	Female
17	Lung	pT2N2M1	IVB	4	44	Female
18	Pancreas	cT3N0M1	IV	4	51	Male
19	Gastric	pT3N1M0	IIIC	6	50	Male
20	Gastric	pT4N3M0	IIIC	3	73	Female
21	Gastric	pT3N3M1	IV	1	68	Male
22	Gastric	pT4N3M1	IV	1	65	Male
23	Gastric	pT3N2M0	IIIA	5	59	Male
24	Gastric	pT4aN3bM0	IIIC	5	50	Male
25	Gastric	pT3N0M0	IIA	8	61	Female
26	Gastric	pT4N2M1	IV	7	40	Female
27	Breast	-	-	Pretreatment	78	Female
28	Breast	pT1N0M0	IA	3	56	Female
29	Breast	pT2N0M0	IIA	5	73	Female
30	Breast	pT1N0M0	IA	6	46	Female
31	Breast	pT2N3M1	IV	5	51	Female

## Data Availability

The data used to support the findings of this study are included within the article.

## Supplementary Materials

The following are available online at https://www.mdpi.com/article/10.3390/cancers14030818/s1, Figure S1: Detailed procedures of image-based algorithmic analysis, Figure S2: Correlation analysis of patient-derived cell clusters with treatment cycles, Figure S3: Correlation analysis of patient-derived cell clusters with gastric cancer treatment cycles, Figure S4: Correlation analysis of patient-derived cell clusters with breast cancer treatment cycles, Figure S5: Correlation analysis of patient-derived cell clusters with T staging using gastric cancer samples under all the treatment cycles, Figure S6: Correlation analysis of patient-derived cell clusters with N staging using gastric cancer samples under all the treatment cycles, Figure S7: Correlation analysis of patient-derived cell clusters with overall cancer staging using gastric cancer samples under all the treatment cycles. Figure S8: Representative images of live/dead (Calcein-AM; green/SYTOX Red; red) staining of patient-derived cell clusters after culturing 14 days. Table S1: Comparison of LIQBP with other existing techniques.

## Abbreviations

TA: thickness per area, LIQBP: liquid biopsy, ROI: region of interest, cROI: cell-based region of interest, nGV: normalized gray value, nSD^GV^: normalized standard deviation of gray value, RGVSD: ratio of the normalized gray value to the normalized standard deviation of clusters, RGVS: ratio of grayscale value to cluster size, PDMS: polydimethylsiloxane, PLA: polylactic acid, DMEM: Dulbecco’s Modified Eagle Medium, FBS: Fetal Bovine Serum.
