# Supplementary material for: Early Predictor Tool of Disease Using Label-Free Liquid Biopsy-Based Platforms for Patient-Centric Healthcare
Source: Cancers (Basel). 2022 Feb 6;14(3):818. doi: 10.3390/cancers14030818 (PMC8834418; doi:10.3390/cancers14030818)
Supplement: Supplementary file 1 [file cancers-14-00818-s001.zip › cancers-1520351-supplementary.pdf]

Supplementary Material

Early predictor tool of disease using label-free liquid biopsy-based platforms for patient-centric healthcare

Wei Li [1,2,†], Yunlan Zhou [3,†] *, Yanlin Deng [1] and Bee Luan Khoo [1,2,4,*]

[1] City University of Hong Kong, 83 Tat Chee Avenue, Kowloon, Hong Kong, 999077, China;

[2] Hong Kong Center for Cerebro-Cardiovascular Health Engineering (COCHE), Hong Kong, 999077, China

[3] Department of Clinical Laboratory, Xinhua Hospital, Shanghai Jiaotong University School of Medicine, Shanghai, 200092, China

[4] City University of Hong Kong Shenzhen Research Institute (CityUSRI), Shenzhen, 518057, China

* Correspondence: cloudsgrace@hotmail.com, blkhoo@cityu.edu.hk

† These authors contributed equally to this work.

Supplementary Figures

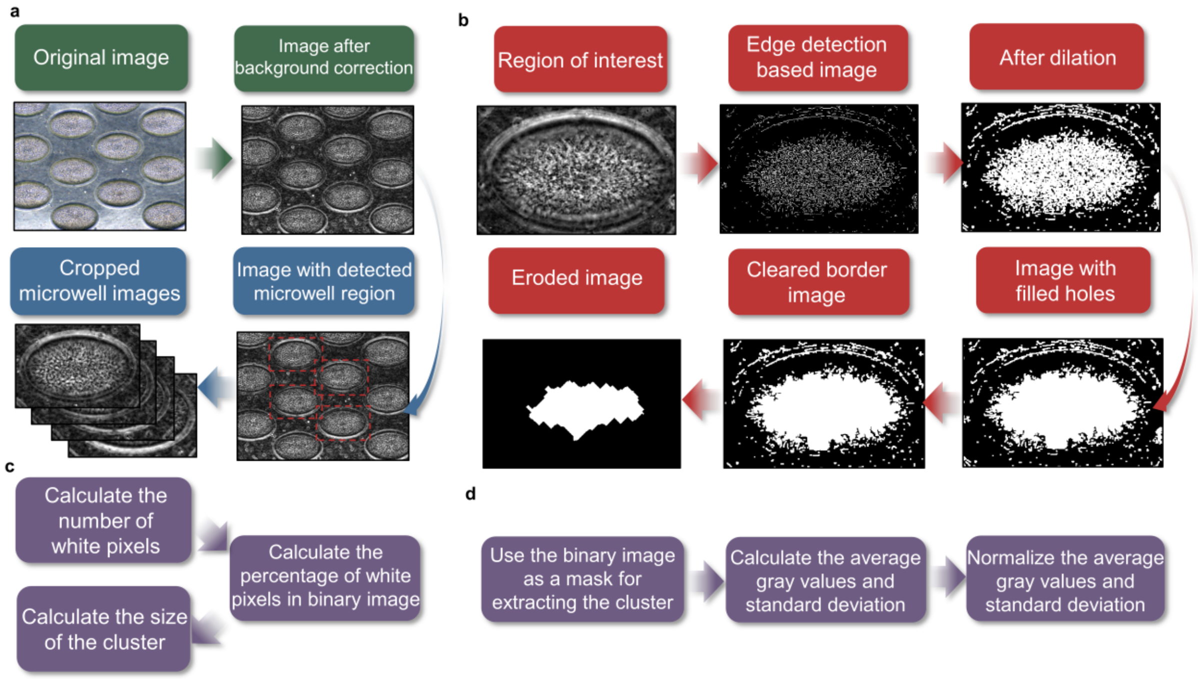

Figure S1. Detailed procedures of image-based algorithmic analysis. (a) ROI feature extraction: The test image was normalized by background correction, and microwells were detected by auto ellipse detection, followed by cropping. (b) Clusters identification: Edges in the cropped image were detected based on the Sobel operator and then transformed into binary features based on thresholding. The binary image was dilated, filled, and eroded sequentially to be used as a mask for feature extraction. (c) Detailed flowchart for cluster size calculation. Cluster size was calculated based on the percentage of white pixels and the scale of the microwell (250 μm × 150 μm). (d) Detailed flowchart for nGV of clusters calculation. The average and highest gray values and nSD^{GV} in the masked image were calculated. The average gray value was normalized to the highest gray value to generate nGV.

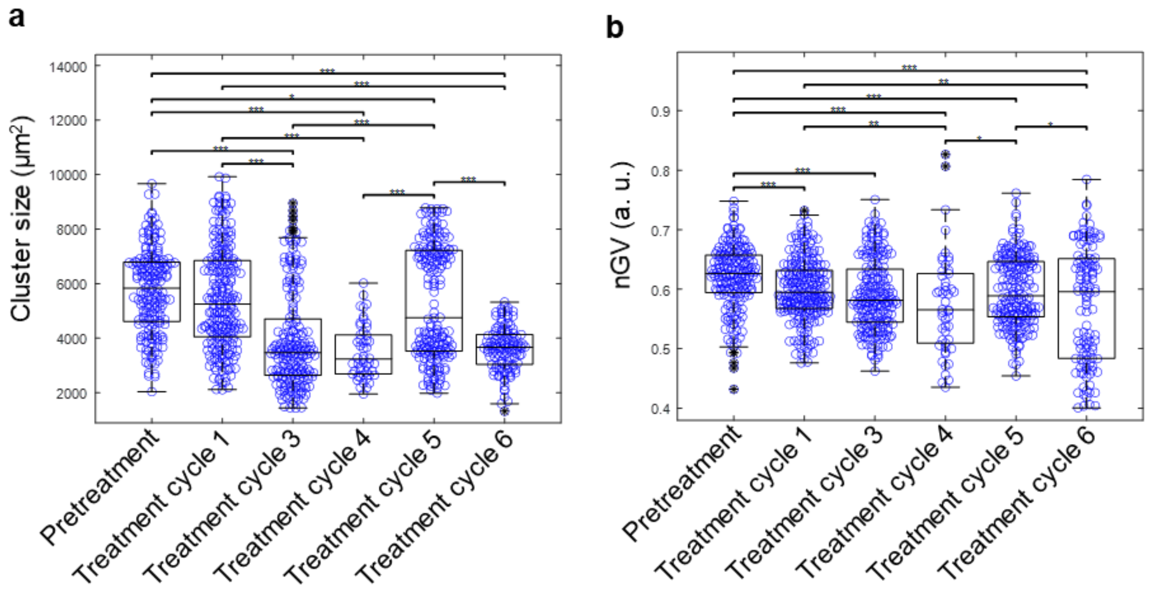

Figure S2. Correlation analysis of patient-derived cell clusters with treatment cycles. (a) and (b) Boxplots of cluster size and nGV of patient clusters (n = 22) versus treatment cycles (pre-treatment (n = 4), treatment cycle 1 (n = 9), treatment cycle 3 (n = 3), treatment cycle 4 (n = 2), treatment cycle 5 (n = 2) and treatment cycle 6 (n = 2)), respectively. *** represents $p \leq 0.001$, ** represents $p \leq 0.01$, and * represents $p \leq 0.05$.

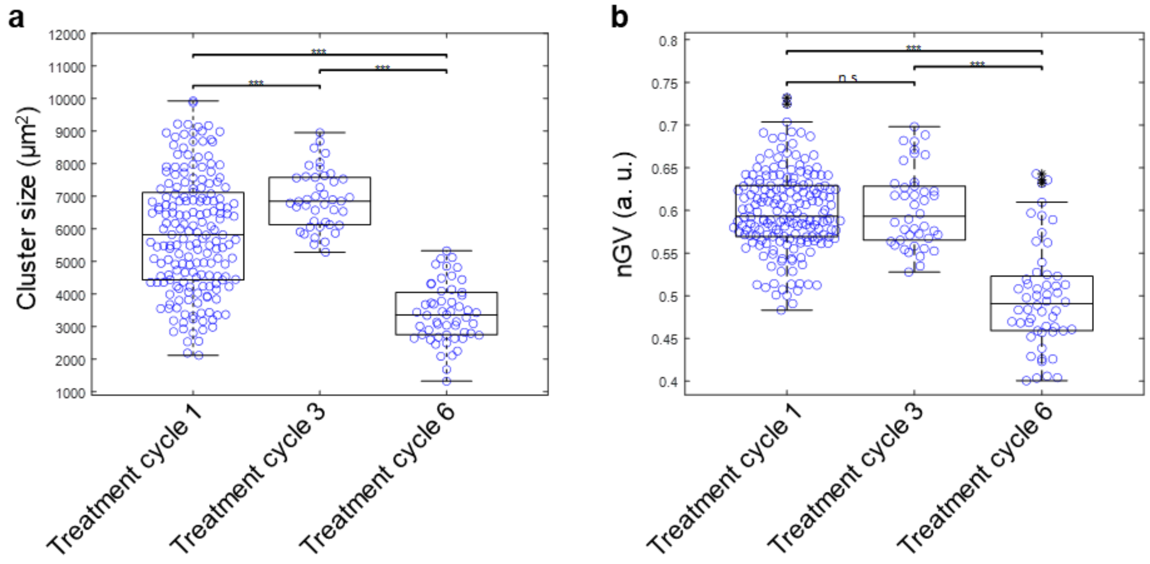

Figure S3. Correlation analysis of patient-derived cell clusters with gastric cancer treatment cycles. (a) and (b) Boxplots of cluster size and nGV of patient clusters from gastric cancer patients (n = 8) versus treatment cycles (treatment cycle 1 (n = 6), treatment cycle 3 (n = 1) and treatment cycle 6 (n = 1)), respectively. *** represents $p \leq 0.001$ and n.s. represents $p > 0.05$.

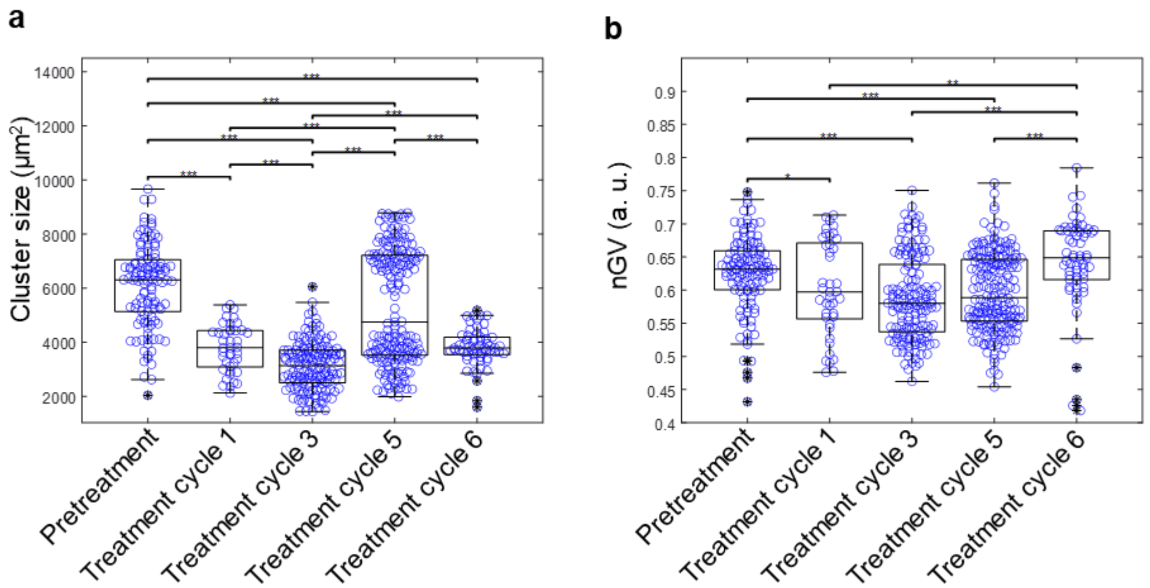

Figure S4. Correlation analysis of patient-derived cell clusters with breast cancer treatment cycles. (a) and (b) Boxplots of cluster size and nGV of patient clusters from breast cancer patients (n = 10) versus treatment cycles (pretreatment (n = 2), treatment cycle 1 (n = 3), treatment cycle 3 (n =2), treatment cycle 5 (n =2) and treatment cycle 6 (n =1)), respectively. *** represents $p \leq 0.001$, ** represents $p \leq 0.01$, and * represents $p \leq 0.05$.

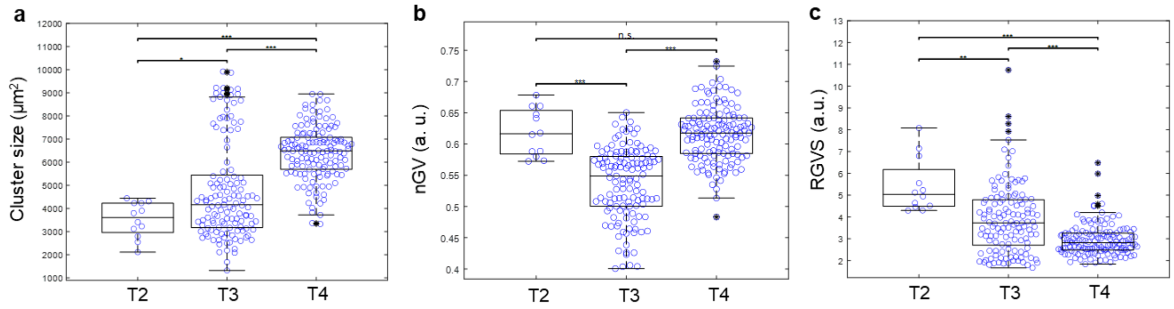

Figure S5. Correlation analysis of patient-derived cell clusters with T staging using gastric cancer samples under all the treatment cycles. (a), (b) and (c) Box plots of size, nGV, and RGVS of the clusters (n = 8) versus T staging, respectively. *** represents $p \leq 0.001$, ** represents $p \leqslant 0.01$, * represents $p \leqslant 0.05$ and n.s. represents $p > 0.05$. T = tumor.

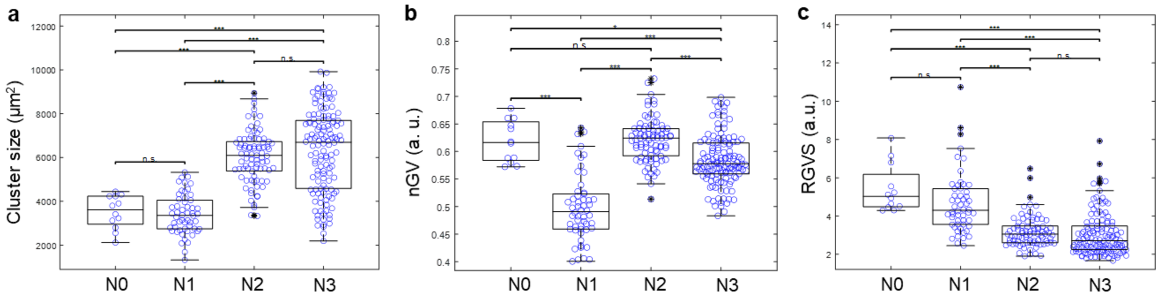

Figure S6. Correlation analysis of patient-derived cell clusters with N staging using gastric cancer samples under all the treatment cycles. (a), (b) and (c) Box plots of size, nGV, and RGVS of the clusters (n = 8) versus N staging, respectively. *** represents $p \leq 0.001$, * represents $p \leq 0.05$ and n.s. represents $p > 0.05$. N = nodes

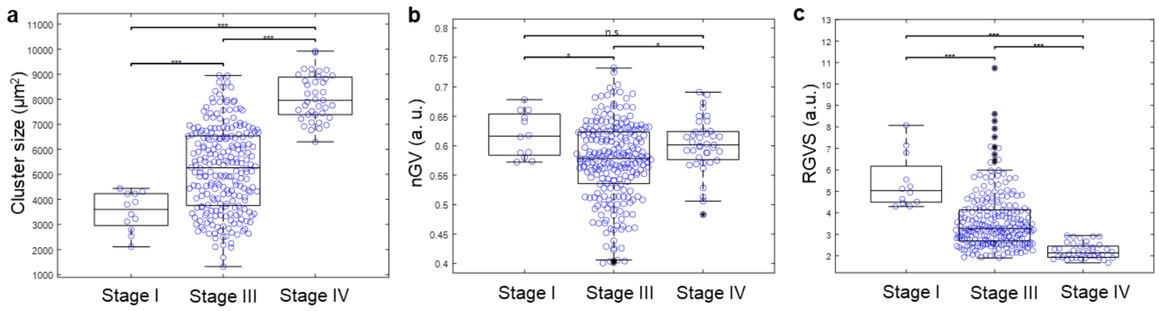

Figure S7. Correlation analysis of patient-derived cell clusters with overall cancer staging using gastric cancer samples under all the treatment cycles. (a), (b) and (c) Box plots of size, nGV, and RGVS of the clusters (n = 8) versus overall cancer staging, respectively. *** represents $p \leq 0.001$, * represents $p \leq 0.05$ and n.s. represents $p > 0.05$.

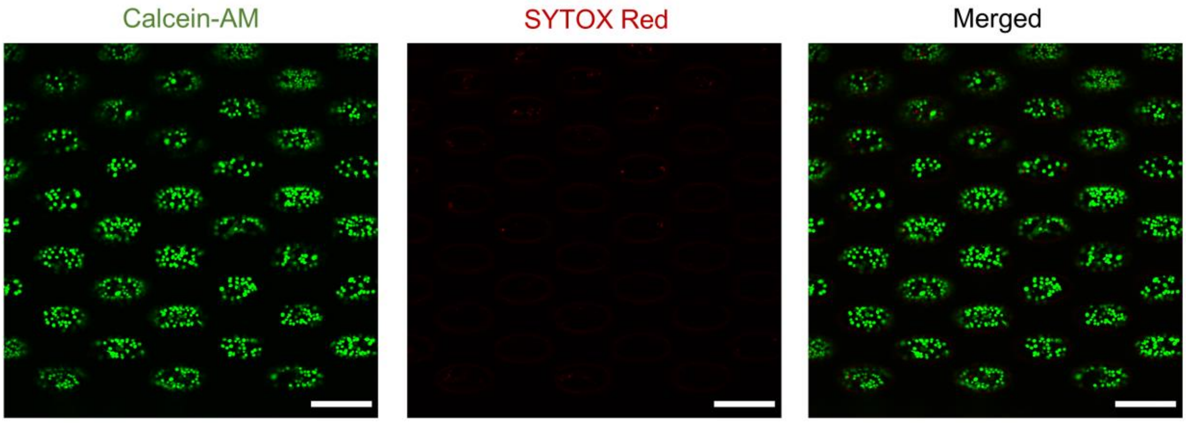

Figure S8. Representative images of live/dead (Calcein-AM; green / SYTOX Red; red) staining of patient-derived cell clusters after culturing 14 days. Scale bars, 250 μm.

Table S1. Comparison of LIQBP with other existing techniques. SEM = scanning electron microscopy, TEM = transmission electron microscopy. NA = not applicable.

**Model type**	**Clusters (LIQBP)**	**Spheroids [1,2]**	**Spheroids [3]**	**Microtissue [4]**	**Spheroids [5]**
Label-free	Yes	Yes	No	Yes	Yes
User interface	Yes	No	No	No	No
Quantitative phenotypes	Cluster size, thickness, roughness, and thickness per area	NA	NA	Fraction of surface area of spheroids	Thickness of basement membrane
Image source	Phase-contrast microscopy	Bright-field microscopy	Fluorescence microscopy	SEM	TEM
System setup	Easy	Medium	Complex	Complex	Complex
Sample type	Clinical samples	Cancer cell line			
Principle	Physics (multi-phenotypes)	Data (viability; formation rate)	Physics (fluorescence intensity)	Physics (fraction of surface area)	Physics (thickness)

References

1. Zhang, Z.; Chen, L.; Wang, Y.; Zhang, T.; Chen, Y.-C.; Yoon, E. Label-Free Estimation of Therapeutic Efficacy on 3D Cancer Spheres Using Convolutional Neural Network Image Analysis. *Analytical chemistry* **2019**, *91*, 14093-14100.
2. Chen, Y.-C.; Zhang, Z.; Yoon, E. Early prediction of single-cell derived sphere formation rate using convolutional neural network image analysis. *Analytical Chemistry* **2020**, *92*, 7717-7724.
3. Goodarzi, S.; Prunet, A.; Rossetti, F.; Bort, G.; Tillement, O.; Porcel, E.; Lacombe, S.; Wu, T.-D.; Guerquin-Kern, J.-L.; Delanoë-Ayari, H. Quantifying nanotherapeutic penetration using a hydrogel-based microsystem as a new 3D in vitro platform. *Lab on a Chip* **2021**.
4. Pitingolo, G.; Nizard, P.; Riaud, A.; Taly, V. Beyond the on/off chip trade-off: A reversibly sealed microfluidic platform for 3D tumor microtissue analysis. *Sensors and Actuators B: Chemical* **2018**, *274*, 393-401.
5. Li, H.; Zheng, Y.; Han, Y.L.; Cai, S.; Guo, M. Nonlinear elasticity of biological basement membrane revealed by rapid inflation and deflation. *Proceedings of the National Academy of Sciences* **2021**, *118*.
